# Normal and melanoma skin visualized, quantified and compared by in vivo photoacoustic imaging

**DOI:** 10.1016/j.pacs.2025.100693

**Published:** 2025-01-29

**Authors:** Terese von Knorring, Tobias Buhl Ihlemann, Paul Blanche, Charlene Reichl, Niels Møller Israelsen, Caroline Meyer Olesen, Yasemin Topal Yüksel, Mette Mogensen

**Affiliations:** a Department of Dermatology, Copenhagen University Hospital, Bispebjerg and Frederiksberg, Copenhagen, Denmark; b Section of Biostatistics, University of Copenhagen, Copenhagen, Denmark; c iThera Medical GmbH, Munich, Germany; d DTU Electro, Department of Electrical and Photonics Engineering, Technical University of Denmark, Kongens Lyngby, Denmark; e Dept of Clinical Medicine, University of Copenhagen, Denmark

**Keywords:** Melanoma, Photoacoustic imaging, Pigmented lesions, Skin cancer, Diagnostics, Noninvasive

## Abstract

Photoacoustic imaging (PAI) shows promise for skin cancer diagnosis by detecting chromophores like melanin, hemoglobin, lipids, and collagen. While most studies focus on malignant lesions, understanding normal skin variability across anatomical regions is crucial for validating PAI's clinical application and its use in melanoma diagnosis. We assessed normal skin in 20 healthy volunteers from three different body locations using a clinical PAI system and compared suspicious looking pigmented skin lesions, including melanomas, to adjacent normal skin (n = 74). Higher deoxyhemoglobin levels were observed in the ankle compared to the cheek and volar forearm, while melanin, lipids, and collagen showed minimal variation. Patients with malignant lesions had significantly higher deoxyhemoglobin levels (p = 0.001) than adjacent normal skin, a difference not seen in benign lesions. These findings suggest that PAI may help diagnose malignancies by identifying increased vascularity in skin cancers, while anatomical differences should be considered during diagnostic work-up.

## Highlights

• Understanding the appearance of normal skin in PAI is crucial for validating its use in skin cancer detection.
• PAI aids skin cancer diagnosis by visualizing skin chromophores.
• PAI identifies increased vascularity in skin cancers compared to normal skin.
• Increased deoxyhemoglobin was found in the ankle compared to the cheek and forearm.
• Hemoglobin and melanin differences across body sites should be considered when quantifying melanin content in skin cancers.

## 1 Introduction

Despite rapid advancements in medical imaging, a significant gap persists in our understanding of normal skin and its transition to malignancy. A deeper understanding of the structure and appearance of normal skin is crucial, serving as a baseline for identifying abnormalities associated with malignant lesions and skin diseases. Adjacent normal skin provides a unique opportunity to investigate whether significant differences exist between lesion and non-lesional skin. Understanding these potential differences could enhance the ability to differentiate between benign and malignant skin tumors, contributing to improved diagnostic precision. Additionally, comparing normal skin at different anatomical sites allows for the establishment of baseline regional variations, providing critical context for interpreting findings in both adjacent and pathological skin.

Photoacoustic imaging (PAI) offers several advancements for skin cancer diagnosis as, it detects and quantifies skin chromophores such as melanin, deoxy- and oxyhemoglobin, lipids and collagen[1]. These differences in PAI can also be utilized for margin delineation during skin cancer surgery[2], [3]. Existing studies have predominantly focused on the delineation and diagnosis of malignant lesions using PAI[4], [5], [6], [7], [8]. Importantly, no clinical studies are currently available that specify the characteristics of normal skin or its variations as captured by PAI.

The advantages of PAI, in comparison to other skin imaging techniques, lie in the combination of optical absorption contrast and ultrasonic spatial resolution. This enables high resolution images at up to centimeter levels depth[1], [7]. The incidence of both keratinocyte carcinomas and cutaneous malignant melanoma (hereafter referred to as melanoma) have been rising over the past decades, thereby increasing patient concern as well as diagnostic workload on doctors [9], [10]. Currently the diagnosis is based upon clinical evaluation of the skin lesion, followed by surgical excision and histopathology analysis[11]. It is recommended to excise the whole lesions when suspicion of melanoma rises [12], [13]. Benign lesions may mimic melanoma, and consequently this course of action may result in numerous costly, unnecessarily excised benign pigmented lesions[13].

We have previously demonstrated that PAI can be used to differentiate malignant (cancerous) from benign (harmless) pigmented skin lesions based on difference in chromophore content and distribution of melanin and blood vessels[8], [14]. Building upon this work, we hypothesize that analyzing adjacent normal skin and its chromophore profiles will help determine whether significant differences exist between lesion and non-lesion skin. Understanding these potential differences could enhance the ability to differentiate between benign and malignant skin tumors. Additionally, examining normal skin from different anatomical regions will enhance our understanding of baseline regional variability. This information is essential to establishing a reference for normal skin characteristics in PAI.

Our objective is to explore normal skin using the unique capabilities of PAI to refine diagnostic precision for skin cancer. Specifically, we aim to: (I) describe chromophore distributions in normal skin across three different anatomical regions: cheek, arm and ankleand (II) compare adjacent PAI data from normal-appearing skin to malignant and benign pigmented lesions. By addressing these gaps, we aim to validate PAI as a reliable tool for skin cancer diagnosis and and set the stage for its potential broader clinical implementation.

## 2 Material and methods

### 2.1 Study design

The data for this study were collected in two separate clinical trials conducted at the Department of Dermatology, Bispebjerg and Frederiksberg University Hospitals, Copenhagen, Denmark. The first study consecutively included patients from May 1 to July 31, 2022. The study protocol was approved by the Danish National Center for Ethics (nr. 2200972) and registered at ClinicalTrials.gov (NCT05389085). The second study, involving healthy volunteers, was conducted in August 2022, and was approved by the Regional Board of Data Collection (nr. P-2022–527). Written consent was obtained from both healthy volunteers and patients enrolled in the two studies, in accordance with the Declaration of Helsinki.

The first study included 75 patients with suspicious pigmented skin lesions with a tentative diagnosis of melanoma, referred from dermatologist and general practitioners to a University Hospital for a second opinion. The lesion, and the adjacent normal skin, were scanned with PAI in a 20–30-minute session. Subsequently, all lesions were excised and sent for histopathology examination, except for one nevus, clinically considered benign at the 3-month follow-up, and one clinically evident dermatofibroma. The results from the PAI data from skin lesions are published in a recent research letter, but data on adjacent normal skin were not included in the data analysis[14]. In the second study 20 healthy volunteers had PAI scans taken from their cheek, volar forearm, and ankle to compare the absorbance of skin chromophores in different anatomical sites. The three anatomical sites, cheek, volar forearm, and ankle were selected to represent a range of skin types and properties. The cheek is a sun-exposed area with relatively thin skin, rich in hair follicles and sebaceous glands. The volar forearm represents sun-exposed, non-hairy skin. The ankle has thicker skin with higher vascular pressure and is less exposed to sunlight, providing a comparison to sun-exposed areas. Gender, age, and Fitzpatrick skin type (FST) were documented in both studies. The Fitzpatrick skin type was assessed clinically and by asking patients about their tendency to burn in the sun at the time of study inclusion. Additionally, skin color measurements with a skin colorimeter were documented for all 20 healthy volunteers. PAI and colorimeter measurements were taken consecutively from the same spot to ensure the same skin area was scanned.

The PAI device used in both studies was a commercially available Multispectral Optoacoustic Tomography (MSOT) Acuity with a 3D probe (iThera Medical GmbH, Munich Germany)[15]. The 3D probe, as shown in Supplement figure 7, is equipped with a transducer with 256 elements, an angular coverage of 120° and a center of frequency of 8 MHz. The probe is filled with D2O as coupling medium between the transducer and the front membrane. The scanning was performed using ultrasound gel with the probe applied directly on the skin, without the use of standoff pad. Skin color in the 20 healthy volunteers was measured using a commercial DermaLab Combo colorimeter (2019 model, Cortex Technology, Denmark) [16]. The device measures skin color using tristimulus colorimetry and narrow-band spectrophotometry, providing continuous values for pigmentation (melanin index, MI) and vascularity (erythema index, EI) with CIE L*a*b* values. The MI calculation is based on the diffuse reflectance in the red spectrum centered at 680 nm[17], [18].

### 2.2 Image analysis

PAI images were acquired at multiple wavelengths (660, 680, 715, 730, 760, 800, 850, 920, 1000, 1064, 1100 and 1210 nm) to unmix specifically for melanin, lipid, collagen, oxy- (HbO2), and deoxyhemoglobin (Hb) based on their unique absorption spectra. A Region of Interest (3D volume) was drawn where melanin signal was present in the lesion skin scans, and the same 3D volume was used for comparison of melanin, lipid, collagen, and blood vessel content in adjacent normal skin scans. In the skin scans from the cheek, forearm, and foot of the healthy volunteers, a 3D volume was drawn as a cubic box with dimensions of 1x1x1 cm in all three anatomical regions, ensuring a uniform volume across the regions. The variables were averaged over the entirety of each 3D volume, and the chromophore content was compared between the three locations.

### 2.3 Statistical method

Six quantitative PAI variables are investigated: melanin concentration, total hemoglobin (HbT), oxyhemoglobin (HbO2), deoxyhemoglobin (HbO), collagen concentration, and lipid concentration. Concentrations are measured in PAI arbitrary units (a.u.). Quantitative data from lesion skin were compared to adjacent normal skin using the paired Welch's *t*-test. Spaghetti plots and boxplots were additionally computed to present the data descriptively. P-values of less than 0.05 were considered statistically significant.

Correlation between age and PAI collagen concentration, as well as between PAI melanin concentration and melanin measured with skin colorimeter (melanin index, MI), was analyzed descriptively using a scatter plot, using data from the 20 healthy volunteers.

## 3 Results

In total 75 patients with pigmented skin lesions suspicious of skin cancer (43 females (57 %), 32 males (42 %), mean age 51 years (min=18, max=83)) and 20 healthy volunteers (12 females (60 %) and 8 males (40 %), mean age 43 years (min=26, max= 83) were included in the study. The majority of patient had FST 2 (51/75 in the suspicious lesion group and 15/20 in the healthy volunteer group). The anatomical distribution of the included lesions was as follows: head and neck (nose, lip, periorbital region, temple, cheek), n = 12/75 (16 %); trunk (scapula, thorax, mamma, abdomen, back, pectoralis), n = 33/75 (44 %); upper limb (upper arm, forearm, hand), n = 6/75 (8 %); lower limb (thigh, crus, knee, sole of foot, toe, ankle), n = 21/75 (28 %); and buttocks (nates), n = 3/75 (4 %). Due to missing scan of adjacent normal skin in one patient, 74 lesions and adjacent normal skin were eligible for analysis, including 18 melanomas and 2 basal cell carcinomas (BCC), 35 melanocytic naevi, and 19 other types of benign skin tumors[14]. The 20 healthy volunteers had PAI scans taken of their cheek, volar forearm, and ankle, resulting in a total of 60 series of PAI images for normal skin analysis.

### 3.1 Pigmented lesions versus normal adjacent skin in PAI

**Fig. 1:**
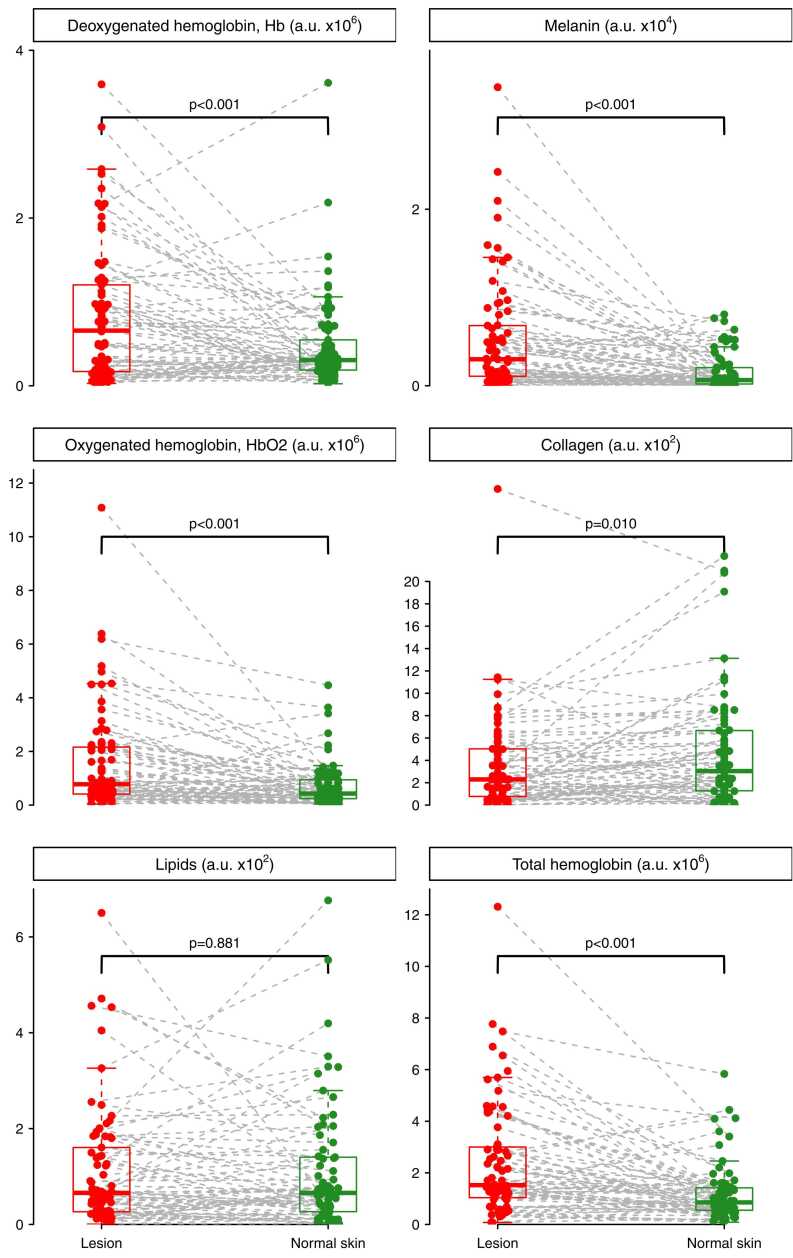
**Multispectral optoacoustic tomography (MSOT) of potential skin cancer compared to normal skin**. Evaluation of in vivo PAI images of patients with potential melanomas and their adjacent normal skin (n = 74). Chromophores including deoxygenated-, oxygenated- and total hemoglobin, lipids, melanin and collagen are compared in two columns, red representing potential melanoma skin lesions versus green representing the patient’s adjacent normal appearing skin. A.u.= MSOT arbitrary unit.

**Fig. 2:**
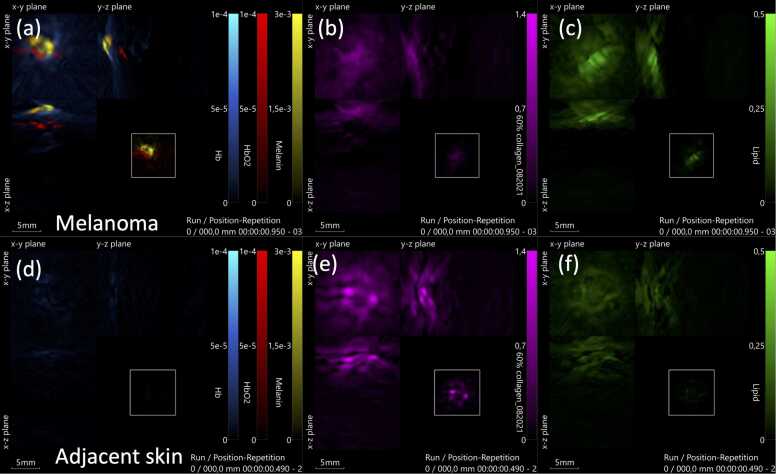
**Melanoma and adjacent normal skin visualized by multispectral optoacoustic tomography (MSOT).** (a-c) Superficially spreading malignant melanoma imaged in vivo in a patient: (a) showing a central melanin signal (yellow) and an abundance of blood vessels, with oxygenated hemoglobin (HbO2) displayed in red and venous blood (Hb) shown in blue, (b) collagen in the lesion, and (c) lipids in the lesion. (d-f) Adjacent normal skin in the same patient: (d) the signals from Hb, HbO₂, and melanin are too low to be visualized, as indicated by the nearly black image, (e) collagen, and (f) lipids, illustrating more collagen and less lipids than the lesion.

Lesional skin, including both benign and malignant lesions, exhibited significantly larger mean values of melanin, deoxy-, oxy and total hemoglobin, and collagen in comparison to adjacent normal skin (p ≤ 0.01) (Fig. 1, Fig. 2). No difference was found in lipid concentration between lesion and adjacent skin.

**Fig. 3:**
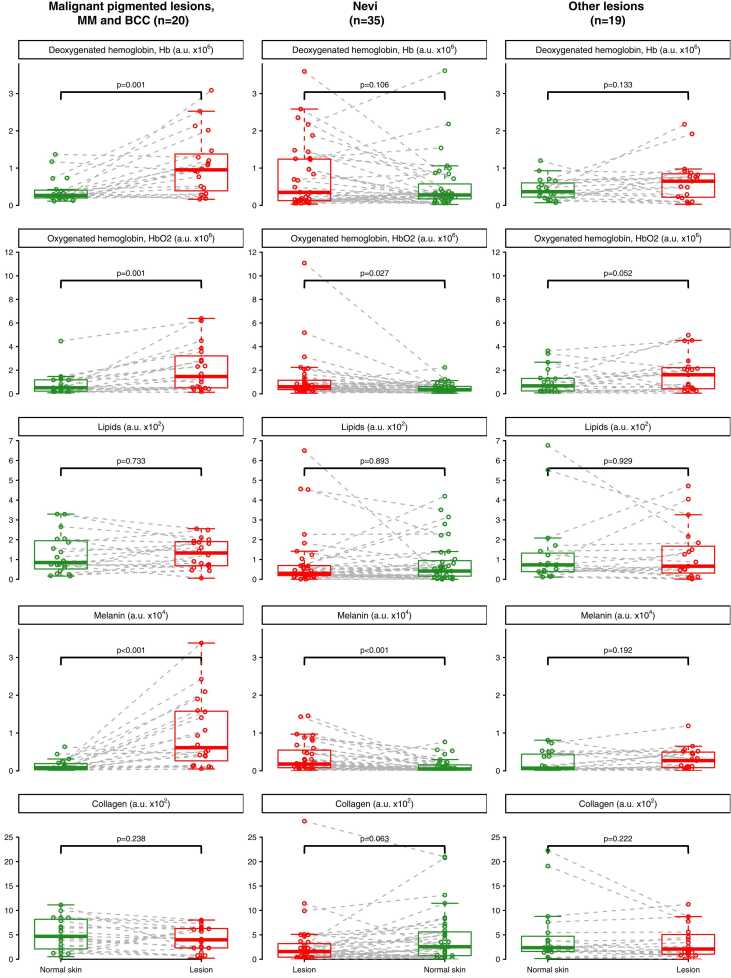
**Comparison of multispectral optoacoustic tomography (MSOT) chromophore concentrations in adjacent normal skin versus skin lesions.** The lesions are categorized into three groups: skin cancer (malignant pigmented lesions, including malignant melanoma [MM] and basal cell carcinoma [BCC]), nevi (pigmented moles), and other benign melanoma mimickers. The boxes illustrate differences in the concentrations of deoxygenated hemoglobin (Hb), oxygenated hemoglobin (HbO2), melanin, lipids, and collagen between normal skin (green dots) and skin lesions (red dots). A.u. = MSOT arbitrary unit.

Comparing malignant lesions to adjacent normal skin, we observed a statically significant difference in deoxyhemoglobin (p = 0.001), which was not found in nevi or other benign lesions compared to adjacent normal skin (Fig. 3). A statistically significant difference was also present for oxyhemoglobin; however, the difference was seen in both malignant lesions/adjacent skin (p = 0.001) and nevi/adjacent skin (p = 0.027). Melanin concentration was significantly higher in both malignant lesions/adjacent skin (p < 0.001) and nevi/adjacent skin (p < 0.001). The difference in collagen concentration appeared more significant in nevi/adjacent skin (p = 0.063) than in malignant lesions/adjacent skin (p = 0.238). Lipids showed no statistically significant difference between either malignant lesions/adjacent skin, or benign lesions/adjacent skin.

### 3.2 Normal skin at different body sites in PAI

**Fig. 4:**
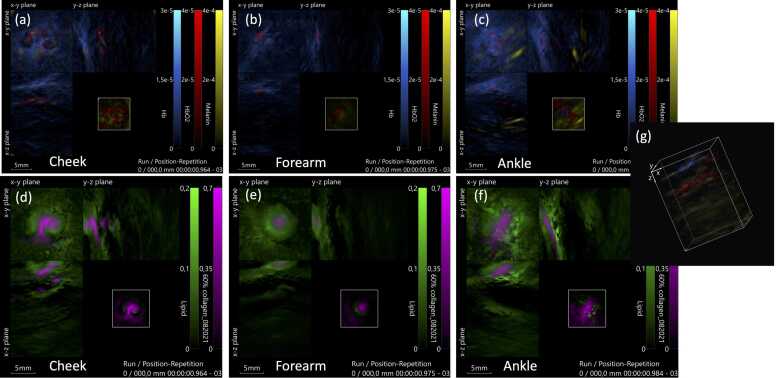
**Normal skin from cheek, forearm and ankle visualized by multispectral optoacoustic tomography (MSOT)**. (a-c) deoxygenated hemoglobin, Hb (blue), oxygenated hemoglobin, HbO2 (red), and melanin (yellow) in cheek (a) forearm (b) and ankle (c). (d-e) collagen (purple) and lipid (green) in cheek (d) forearm (e) and ankle (f). The concentration of hemoglobin, especially deoxygenated hemoglobin (blue), appeared to be higher in the ankle than the forearm and cheek. The concentration of melanin, lipids and collagen showed minimal variation among the cheek, forearm, and ankle.

**Fig. 5:**
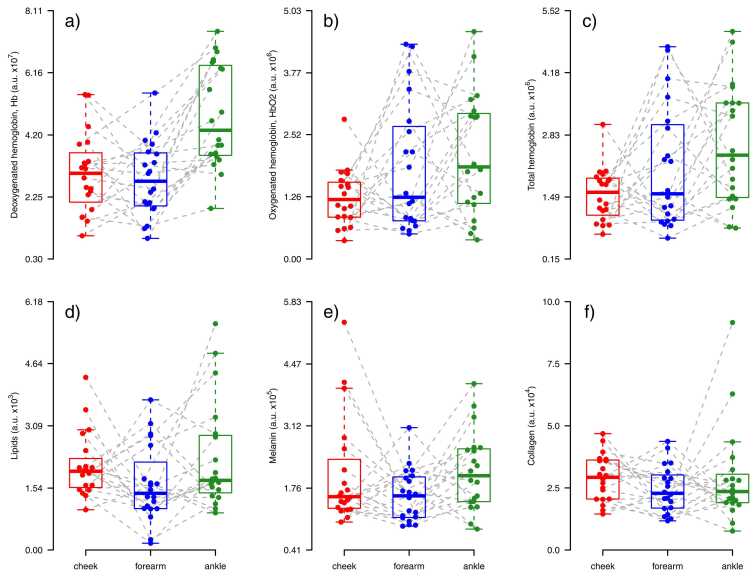
**Normal skin in different body locations.** Multispectral optoacoustic tomography (MSOT) measured concentration of chromophores in healthy volunteers (n = 20). The chromophores shown are (a) deoxygenated hemoglobin, (b) oxygenated hemoglobin, (c) total hemoglobin, (d) lipids, (e) melanin and (f) collagen in three anatomical sites: cheek (red), volar forearm (blue), and ankle (green). A.u= MSOT arbitrary unit.

When comparing the different anatomical sites (cheek, volar forearm, and ankle) in the 20 healthy volunteers, the concentration of both oxy-, deoxy- and total hemoglobin, appeared to be higher in the ankle than the forearm and cheek (Fig. 4, Fig. 5). The medians of deoxyhemoglobin in the ankle compared to the cheek and forearm were 45 % and 59 % higher, respectively. The concentration of melanin, lipids and collagen showed minimal variation among the cheek, forearm, and ankle.

No correlation was observed between MSOT melanin concentration and melanin index measured with skin colorimeter from cheek, forearm, and ankle, nor between age and MSOT collagen concentration among the 20 healthy volunteers (Supplementary Fig.6).

## 4 Discussion

There is limited data on skin chromophores in normal human skin using PAI technology. Studying skin chromophores may be key to understanding skin disease and malignancy transformation. To accurately interpret results from skin tumors, it is essential to establish a baseline by understanding how normal skin appears in PAI across different body sites and skin colors.

It is well known that the formation of new blood vessels, or angiogenesis, is a highly malignant trait, playing a major role in tumor progression[19], [20], [21]. We found a significant increase in venous blood flow in malignant lesions compared to adjacent normal skin, showcased by deoxyhemoglobin, a difference not found in either nevi or other benign lesions (Fig. 3). This corresponds to previous findings where the concentration of deoxyhemoglobin was significantly higher in malignant lesions compared to benign lesions[8], [14]. Arterial blood flow, indicated by oxyhemoglobin, was significantly increased in malignant lesions compared to adjacent normal skin. However, this increase was also observed in benign lesions compared to adjacent normal skin, and therefore, it was not specific to malignancy. This suggests that, in a clinical setting, an elevation in the concentration of oxyhemoglobin alone in PAI may not be sufficient to distinguish a malignant skin lesion from a benign one.

We observed a significant difference in melanin concentration between all lesions and normal adjacent skin (Fig. 1, Fig. 2). Both malignant lesions and nevi had significantly higher levels of melanin compared to the corresponding adjacent skin, whereas other benign lesions did not show a significant difference (Fig. 3). This finding aligns with the fact that melanocytes are present in both nevi and malignant melanoma, emphasizing that increased melanin concentration alone may not be useful for differentiating between cancer and a harmless nevus (mole)[22].

The role of skin lipids in healthy, as well as diseased, human skin, have been studied and quantified in both the stratum corneum and other parts of epidermis[23], [24]. Knowledge of the concentration of collagen and lipids is important when comparing normal skin to malignant lesions. Skin cancers, especially melanoma, have been described to contain more collagen [25], [26], [27] and an increased rate of lipid synthesis have been described in both melanoma and non-melanoma skin cancers [28], [29], [30]. Previously, we found higher concentrations of lipids and collagen in PAI in malignant lesions compared to benign[14]. Despite this, we found no difference in lipid concentration between lesional skin and adjacent normal skin, whether in malignant lesions or benign lesions. Interestingly, the collagen concentration seemed to be increased (p = 0.063) in nevi compared to adjacent normal skin, but not in malignant lesions. In general, the concentration of collagen was significantly higher in lesions compared to adjacent normal skin (Fig. 1).

Higher concentrations of especially deoxyhemoglobin were found in the ankle compared to the cheek and forearm, which corresponds with the fact that the ankle is an anatomical site with an abundance of blood vessels, including many intertwining subdermal plexuses of arterioles and venules, and a higher venous pressure[31]. Surprisingly, the differences in melanin, collagen, and lipid concentrations measured with PAI showed little variance between the cheek, forearm, and ankle. Since melanin is produced endogenously in melanocytes to protect the skin in response to UV damage, we would expect higher melanin concentrations in the face, being the most sun-exposed area of the body[32]. Collagen, on the other hand, has been shown to decrease in response to UV damage, as it is degraded by UV rays[33]. Yet, we found that collagen concentrations showed very little difference among the cheek, forearm, and ankle (Fig. 4, Fig. 5). We also anticipated finding a negative correlation between collagen concentration and age, as aging is known to be linked with a reduction in collagen fibers in the skin [34]. However, this was not the case (Supplementary Fig.6b), which may be due to the small sample size with few representatives in each age group.

We chose to compare colorimeter measured MI with PAI measured melanin concentration, as FST relies on self-assessed subjective assessment of skin type prone to misclassification, while MI is an objective value[35], [36], [37]. Skin color influences how light interacts with the skin, affecting its absorption and scattering [38], [39]. Understanding these effects is essential to ensure that light-based medical tests and treatments work effectively for all skin tones. We hypothesized a positive correlation between PAI melanin concentration and MI measured with skin colorimeter; however, no such correlation was observed (Supplementary Fig. 6a). Empirical data from literature suggests that light penetrates skin more easily at wavelengths above 940 nm. As the wavelength increases, the amount of light transmitted through the skin becomes more similar across different skin colors[40]. This could explain the lack of variance in melanin concentration among our patients. Importantly, most participants in our study had light skin (FST 2),so the differences from one subject to another may be too subtle to be apparent in PAI and colorimeter measures. Further PAI research is needed to determine how darker skin types might impact these results.

## 5 Conclusion

Understanding how normal skin appears in PAI across different body sites and adjacent to skin cancers or other lesions is crucial for interpreting PAI scan results in skin diseases. This knowledge is key to evaluating the diagnostic potential of PAI in a dermatology clinic. Our study found higher levels of especially deoxyhemoglobin in the ankle compared to the cheek and volar forearm. Melanin, lipids and collagen showed minimal variation between these body sites. Importantly, deoxyhemoglobin was significantly higher in malignant skin lesions compared to adjacent normal skin; a difference not observed in benign lesions. These findings suggest that PAI could be a valuable tool in diagnosing malignancy by identifying increased vascularity in skin cancers, while differences in melanin and hemoglobin at different body sites should be considered. Further research could test different thresholds for a positive melanoma diagnosis using PAI data, requiring a much larger sample size.

## Funding sources

This study was funded by unrestricted grants from Vissing Foundation, Axel Muusfeldt Foundation, Toyota- Foundation Denmark, Arvid Nilsson´s Foundation, NEYE Foundation, Aase and Ejnar Danielsens Foundation, Koebmand Sven Hansen & Hustru Ina Hansen´s Foundation, and The Danish Cancer Society.

## CRediT authorship contribution statement

**Buhl Ihlemann Tobias:** Writing – review & editing, Visualization, Formal analysis, Data curation. **Reichl Charlene:** Writing – review & editing, Visualization, Methodology, Formal analysis, Data curation. **Blanche Paul:** Writing – review & editing, Visualization, Methodology, Formal analysis, Data curation. **Mogensen Mette:** Writing – review & editing, Supervision, Resources, Project administration, Investigation, Funding acquisition, Formal analysis, Conceptualization. **Topal Yuksel Yasemin:** Writing – review & editing, Methodology, Investigation, Data curation. **Meyer Olesen Caroline:** Writing – review & editing, Methodology, Investigation. **Møller Israelsen Niels:** Writing – review & editing, Supervision, Methodology. **von Knorring Terese:** Writing – original draft, Visualization, Methodology, Investigation, Funding acquisition, Formal analysis, Data curation, Conceptualization.

## Declaration of Competing Interest

The authors declare that they have no known competing financial interests or personal relationships that could have appeared to influence the work reported in this paper.

## Data Availability

The data that has been used is confidential.
