# Supplementary material for: Normal and melanoma skin visualized, quantified and compared by in vivo photoacoustic imaging
Source: Photoacoustics. 2025 Jan 29;42:100693. doi: 10.1016/j.pacs.2025.100693 (PMC11836482; doi:10.1016/j.pacs.2025.100693)
Supplement: Supplementary file 1 — Supplementary material [file mmc1.docx]

**Figure 1.** **Multispectral optoacoustic tomography (MSOT) of potential skin cancer compared to normal skin**. Evaluation of in vivo PAI images of patients with potential melanomas and their adjacent normal skin (n=74). Chromophores including deoxygenated-, oxygenated- and total hemoglobin, lipids, melanin and collagen are compared in two columns, red representing potential melanoma skin lesions versus green representing the patient’s adjacent normal appearing skin. A.u.= MSOT arbitrary unit.

**Figure 2.** **Melanoma and adjacent normal skin visualized by multispectral optoacoustic tomography (MSOT).** (a-c) Superficially spreading malignant melanoma imaged in vivo in a patient: (a) showing a central melanin signal (yellow) and an abundance of blood vessels, with oxygenated hemoglobin (HbO2) displayed in red and venous blood (Hb) shown in blue, (b) collagen in the lesion, and (c) lipids in the lesion. (d-f) Adjacent normal skin in the same patient: (d) the signals from Hb, HbO₂, and melanin are too low to be visualized, as indicated by the nearly black image, (e) collagen, and (f) lipids, illustrating more collagen and less lipids than the lesion.

**Figure 3.** **Comparison of multispectral optoacoustic tomography (MSOT) chromophore concentrations in adjacent normal skin versus skin lesions.** The lesions are categorized into three groups: skin cancer (malignant pigmented lesions, including malignant melanoma [MM] and basal cell carcinoma [BCC]), nevi (pigmented moles), and other benign melanoma mimickers. The boxes illustrate differences in the concentrations of deoxygenated hemoglobin (Hb), oxygenated hemoglobin (HbO2), melanin, lipids, and collagen between normal skin (green dots) and skin lesions (red dots). A.u. = MSOT arbitrary unit.

**Figure 4. Normal skin from cheek, forearm and ankle visualized by multispectral optoacoustic tomography (MSOT)**. (a-c) deoxygenated hemoglobin, Hb (blue), oxygenated hemoglobin, HbO2 (red), and melanin (yellow) in cheek (a) forearm (b) and ankle (c). (d-e) collagen (purple) and lipid (green) in cheek (d) forearm (e) and ankle (f). The concentration of hemoglobin, especially deoxygenated hemoglobin (blue), appeared to be higher in the ankle than the forearm and cheek. The concentration of melanin, lipids and collagen showed minimal variation among the cheek, forearm, and ankle.

**Figure 5. Normal skin in different body locations.** Multispectral optoacoustic tomography (MSOT) measured concentration of chromophores in healthy volunteers (n=20). The chromophores shown are (a) deoxygenated hemoglobin, (b) oxygenated hemoglobin, (c) total hemoglobin, (d) lipids, (e) melanin and (f) collagen in three anatomical sites: cheek (red), volar forearm (blue), and ankle (green). A.u= MSOT arbitrary unit

**Supplementary Figure 6. Correlation between multispectral optoacoustic tomography (MSOT) measured melanin concentration and melanin measured with skin colorimeter; and MSOT measured collagen concentration and age in healthy volunteers (n=20).**

a) MSOT melanin concentration in cheek (red), volar forearm (blue) and ankle (green) plotted against melanin measured with skin colorimeter. b) MSOT collage concentration in cheek (red), volar forearm (blue) and ankle (green) plotted against age. MI=melanin index, a.u.= MSOT arbitrary unit

**Supplementary Figure 7. Multispectral optoacoustic tomography (MSOT) 3D probe.** Images of the MSOT Acuity 3D probe with, on the left, the fully mounted probe and, on the right, the transducer**.**
