# Supplementary figures and images for: Normal and melanoma skin visualized, quantified and compared by in vivo photoacoustic imaging
Source: Photoacoustics. 2025 Jan 29;42:100693. doi: 10.1016/j.pacs.2025.100693 (PMC11836482; doi:10.1016/j.pacs.2025.100693)

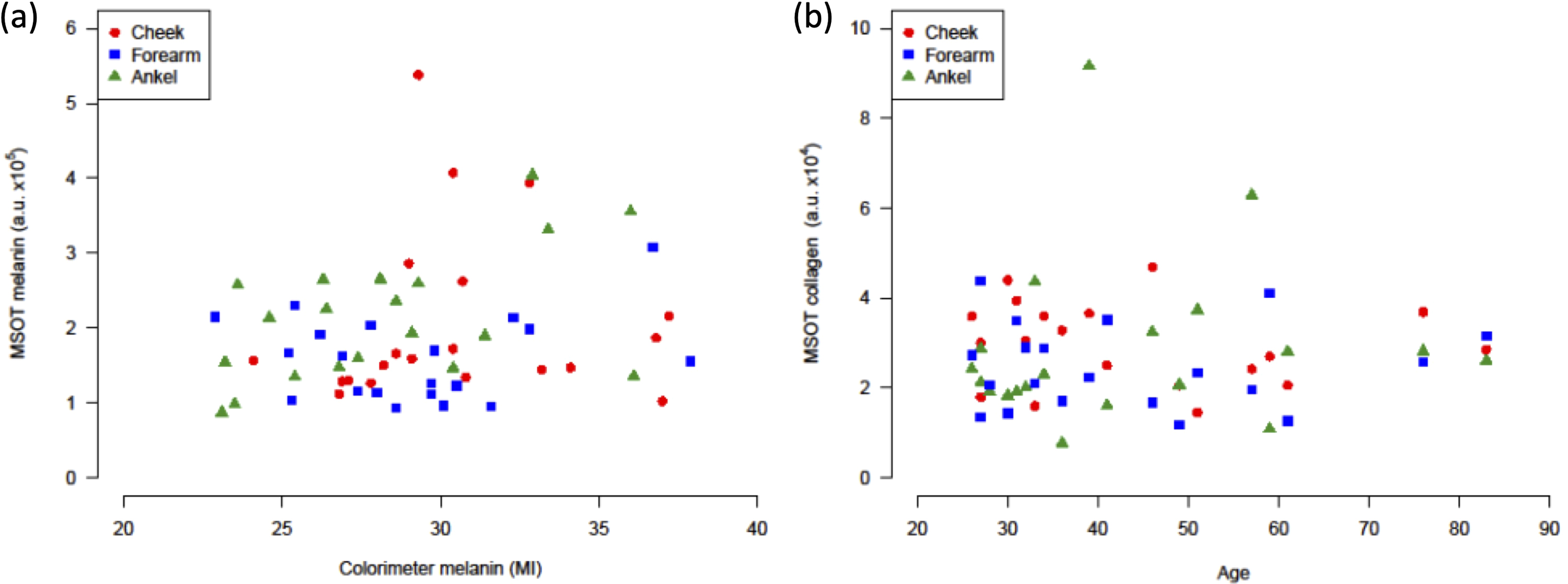

Supplement: Supplementary file 2 — Supplementary material [file mmc2.jpg]

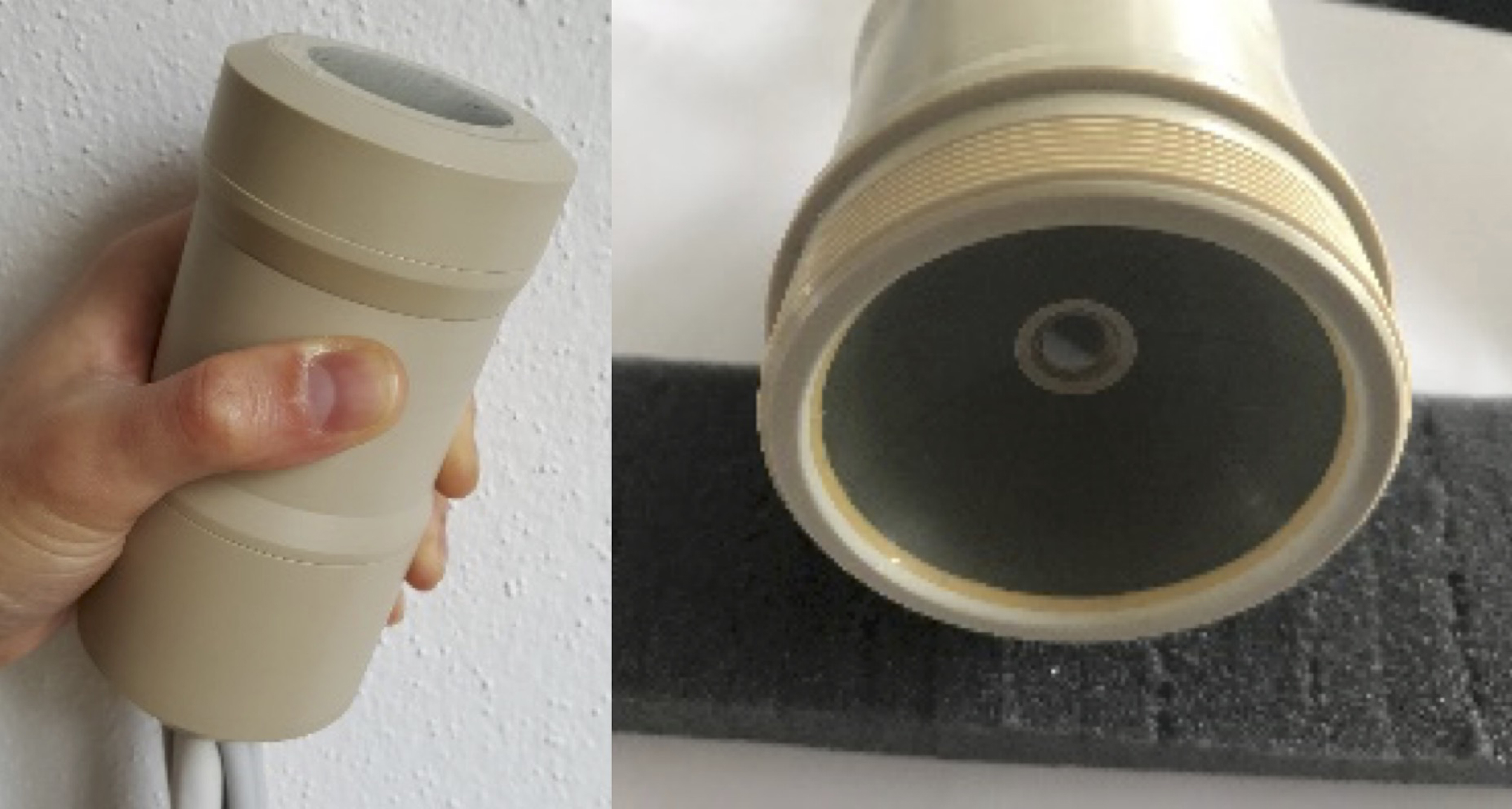

Supplement: Supplementary file 3 — Supplementary material [file mmc3.jpg]
